# Serine 1179 Phosphorylation of Endothelial Nitric Oxide Synthase Increases Superoxide Generation and Alters Cofactor Regulation

**DOI:** 10.1371/journal.pone.0142854

**Published:** 2015-11-11

**Authors:** Hu Peng, Yugang Zhuang, Mark C. Harbeck, Donghong He, Lishi Xie, Weiguo Chen

**Affiliations:** 1 Department of Emergency Medicine, Shanghai Tenth People’s Hospital, Tongji University, Shanghai, China; 2 Division of Pulmonary, Critical Care, Sleep and Allergy, Department of Medicine, University of Illinois College of Medicine, Chicago, Illinois, United States; University of North Dakota, UNITED STATES

## Abstract

Endothelial nitric oxide synthase (eNOS) is responsible for maintaining systemic blood pressure, vascular remodeling and angiogenesis. In addition to producing NO, eNOS can also generate superoxide (O_2_^-.^) in the absence of the cofactor tetrahydrobiopterin (BH4). Previous studies have shown that bovine eNOS serine 1179 (Serine 1177/human) phosphorylation critically modulates NO synthesis. However, the effect of serine 1179 phosphorylation on eNOS superoxide generation is unknown. Here, we used the phosphomimetic form of eNOS (S1179D) to determine the effect of S1179 phosphorylation on superoxide generating activity, and its sensitivity to regulation by BH4, Ca^2+^, and calmodulin (CAM). S1179D eNOS exhibited significantly increased superoxide generating activity and NADPH consumption compared to wild-type eNOS (WT eNOS). The superoxide generating activities of S1179D eNOS and WT eNOS did not differ significantly in their sensitivity to regulation by either Ca^2+^ or CaM. The sensitivity of the superoxide generating activity of S1179D eNOS to inhibition by BH4 was significantly reduced compared to WT eNOS. In eNOS-overexpressing 293 cells, BH4 depletion with 10mM DAHP for 48 hours followed by 50ng/ml VEGF for 30 min to phosphorylate eNOS S1179 increased ROS accumulation compared to DAHP-only treated cells. Meanwhile, MTT assay indicated that overexpression of eNOS in HEK293 cells decreased cellular viability compared to control cells at BH4 depletion condition (P<0.01). VEGF-mediated Serine 1179 phosphorylation further decreased the cellular viability in eNOS-overexpressing 293 cells (P<0.01). Our data demonstrate that eNOS serine 1179 phosphorylation, in addition to enhancing NO production, also profoundly affects superoxide generation: S1179 phosphorylation increases superoxide production while decreasing sensitivity to the inhibitory effect of BH4 on this activity.

## Introduction

Nitric oxide (NO) synthesized by the endothelial NO synthase (eNOS) plays important and diverse roles in numerous biological processes, including vascular tone regulation, vascular remodeling, and angiogenesis [1–3]. Under certain conditions, including BH4 depletion or low cellular [Ca^2+^], eNOS generates superoxide (O_2_^-.^) rather than NO[4], which can in turn give rise to a number of deleterious side effects, including the formation of peroxynitrite (ONOO^-^) [4–6]. Nitric oxide synthase (NOS) activity is regulated at multiple levels, including gene transcription, post-translational modification, and protein-protein interactions [7–8].

Recent studies have shown that phosphorylation of eNOS enhances eNOS activity and alters eNOS enzyme characteristics. Studies of VEGF and shear-stress-mediated phosphorylation of eNOS indicated that the serine/threonine protein kinase Akt/PKB mediates the activation of eNOS, leading to increase NO production [9]. Mimicking the phosphorylation of Ser 1177 directly enhances enzyme activity and alters the sensitivity of the enzyme to Ca^2+^, rendering its activity maximal at sub-physiological concentrations of Ca^2+^ [9–10]. Thus, phosphorylation of eNOS by Akt represents a novel Ca^2+^-dependent regulatory mechanism for activation of eNOS. An important characteristic of eNOS is its capacity to generate O_2_^-.^ in the absence of L-arginine and BH_4_, by the same mechanism used to generate NO [4, 11]. However, it is unclear if Serine 1179 phosphorylation regulates O_2_^-.^ generation by eNOS. It is also not known if O_2_^-.^ generation by eNOS is regulated by Ca^2+^, CaM, and BH_4_ in the same manner as NO generation.

In the current study, we used wild type eNOS (WT eNOS) and a phosphomimetic mutant eNOS (S1179D eNOS), where the aspartate carboxyl side group mimics a negatively charged phosphate group on serine 1179, to investigate the effect of eNOS phosphorylation on O_2_^-.^ generation. Our results indicate that serine 1179 phosphorylation enhances the O_2_^-.^ generating activity of eNOS and alters the affinity of eNOS for cofactors that shift the balance of eNOS activity between NO and O_2_^-.^ generation. Additionally, we show that serine 1179 phosphorylation enhances O_2_^-.^-generating eNOS activity in cells and decreases cell viability.

## Materials and Methods

### Materials

Cell culture medium and growth factor kits were obtained from Gibco BRL (Gaithersburg, MD). 2', 5’-ADP-Sepharose 4B was obtained from Pharmacia Biotech. Inc. (Piscataway, NJ). L-[^14^C]Arginine was purchased from DuPont/NEN (Boston, MA). 5-Diethoxyphosphoryl-5-methyl-1-pyrroline-*N*-oxide (DEPMPO, >99% pure) was purchased from Oxis International Inc. (Portland, OR). Superoxide dismutase (SOD), CaM, NADPH, L-arginine, BH_4_, *N*-nitro-L-arginine methyl ester (L-NAME), and other reagents were purchased from Sigma Chemical Co. (St. Louis, MO), unless otherwise indicated.

### eNOS Purification

Recombinant bovine wild-type eNOS and S1179D eNOS plasmids were purchased from Addgene (Cambridge, MA 02139). eNOS plasmids were over-expressed in E.Coli (BL21), the bacteria were harvested, and then lysed by pulsed sonication in buffer A (50 mM Tris-HCl, pH 7.5, 0.1 mM EDTA, 0.1 nmM dithiothreitol, 150mM NaCl, 10% Glycerol (v/v), 1 mg/ml lysozyme, and bacterial proteinase inhibitor cocktail). The mixture was then treated with DNase (10U/ml) to remove DNA. After centrifugation (100,000 × *g* for 30 min), the supernatant was applied to a 2',5'-ADP-Sepharose 4B column (1.5 × 2 cm) pre-equilibrated in buffer A. The column was washed with 25 ml of buffer A followed by 25 ml of buffer B (50 mM Tris-HCl, pH 7.5, 0.1 mM EDTA, 0.1 mM dithiothreitol, 600mM NaCl, and 10% Glycerol (v/v)). The protein was eluted with 5 mM adenosine 2', 3’-monophosphate in buffer E (50 mM Tris-HCl, pH 7.5, 0.1 mM EDTA, 1 mM dithiothreitol, 600mM NaCl, and 10% glycerol). The eluate was concentrated using a Centriprep 50 (Amicon). Further purification was performed with FPLC. The purified eNOS was selected from the fractions absorbing at 280nm and 408 nm (absorbance by the eNOS heme group). The concentrated eNOS proteins were stored at -80°C in elution buffer with 10% glycerol. Protein concentration was assayed with Bradford reagent (Bio-Rad) using bovine serum albumin as standard [12]. The purity of eNOS was determined by SDS-polyacrylamide gel electrophoresis (SDS/PAGE) and visualized with Coomassie Blue staining.

### L-[14C]arginine to L-[14C]citrulline conversion assay

eNOS-catalyzed L-[^14^C]arginine to L-[^14^C]citrulline conversion was monitored in a total volume of 200 μl of buffer containing 50 mM Tris-HCl, pH 7.4, 2μM L-[^14^C]arginine, 0.5 mM NADPH, 0.5 mM Ca^2+^, 10 μg/ml calmodulin, 2.5 μM BH_4_, and 5 μg/ml purified eNOS. After 5-min incubation at 37°C, the reaction was terminated by adding 3 ml of ice-cold stop buffer (20mM Hepes, pH 5.5,2mM EDTA, 2mM EGTA). L-[^14^C]citrulline was separated by passing reaction mixtures through Dowex AG 50W-X8 (Na^+^ form, Bio-Rad) cation exchange columns and quantified by liquid scintillation counting [4, 13].

### EPR spectroscopy and spin trapping

Spin-trapping measurements of oxygen free radicals were performed in 50 mM Tris-HCl buffer, pH 7.6,containing 0.5 mM NADPH, 0.5 mM Ca^2+^, 10 μg/ml calmodulin, 300nM purified eNOS, and 20 mM spin trap DEPMPO. EPR spectra were recorded in a disposable micropipette (50μl, VWR Scientific) at room temperature (23°C) with a Bruker EMX spectrometer operating at X-band with a high sensitive (HS) cavity (Brucker Instrument, Billerica, MA) using a modulation frequency of 100 kHz, modulation amplitude of 0.5 G, microwave power of 20 milliwatts, and microwave frequency of 9.863GHz, as described [14–15]. The central magnetic field was 3510.0 G, and the sweep width was 140.0 G. Time constant was 163.84miliseconds. Sweep rate was 40.96miliseconds. Receiver gain was 2×10^6^. Spectra were continuously recorded at 1 min acquisition from the beginning of the reaction until 30 min.

### NADPH consumption by eNOS

NADPH oxidation was followed spectrophotometrically at 340 nm [4, 16]. The reaction systems were the same as described in EPR measurements, and the experiments were run at room temperature. The rate of NADPH oxidation was calculated using a molar extinction coefficient of 6.22/mM/cm.

### Cell culture and transfection

Human embryonic kidney (HEK) 293 cells (Sigma Aldrich, St. Louis, MO) were grown in Dulbecco’s modified Eagle’s medium supplemented with10% fetal bovine serum. HEK 293 cells do not express either eNOS mRNA or eNOS protein, and are thus an “eNOS null” cell line. After HEK293 cells were grown to 80% confluence, either empty-vector plasmid or eNOS plasmid DNA were transfected into the cells using lipofectamine 2000, according to the manufacturer’s protocols, followed by the treatments described [17].

### Measurement of intracellular ROS generation in eNOS-overexpressing HEK 239 cells

Detection of intracellular ROS was performed by an established method using the ROS-sensitive fluorescent probe 2',7'-dihydrodichlorofluorescin diacetate (DCF-DA) and fluorescence microscopy. For measurement of intracellular ROS, HEK 293 cells and eNOS overexpressing HEK 239 cells were plated in 24 well plates (Fisher Sciences, Newark, DE) at a density of 2 × 10^4^ cells/ml, and cultured with 10mM DAHP for 2 days. The cells were then cultured with either 1μM A23187 alone, or 1μM A23187 plus 50ng/ml VEGF, for 30 minutes. The cells were washed with Dulbecco’s phosphate-buffered saline (DPBS) and were loaded with 5 μg/ml of DCF-DA (Molecular Probes, Eugene, OR) for 5 min at 37°C. The fluorescent intensity of dichlorofluorescin was quantified by using a fluorescence microscope (Nikon, Phase Contrast 2, Japan), with excitation and emission wavelengths of 488 and 520 nm, respectively [18].

### Cell viability analysis by 3-(4,5-dimethylthiazole)-2,5-diphenyl tetrazolium bromide (MTT) assay

HEK 293 cells and eNOS overexpressing HEK 239 cells were treated with 10mM DAHP for 48 hours, and eNOS activated with 1μM A23187, or 1μM A23187 plus 50ng/ml VEGF, overnight. Following treatment, cellular viability was measured by MTT reduction assay, which is used to assess cell metabolic activity. 40 μl of 5 mg/ml MTT solution was added to each well. After 1h of incubation, the supernatant absorbance of each well was measured spectrophotometrically at 570nm (Cary 50 Bio, UV-Visible Spectrophotometer) according to the manufacturer's protocol [19–20].

## Results

### S1179D eNOS has more NO-generating activity than WT eNOS

**Fig 1:**
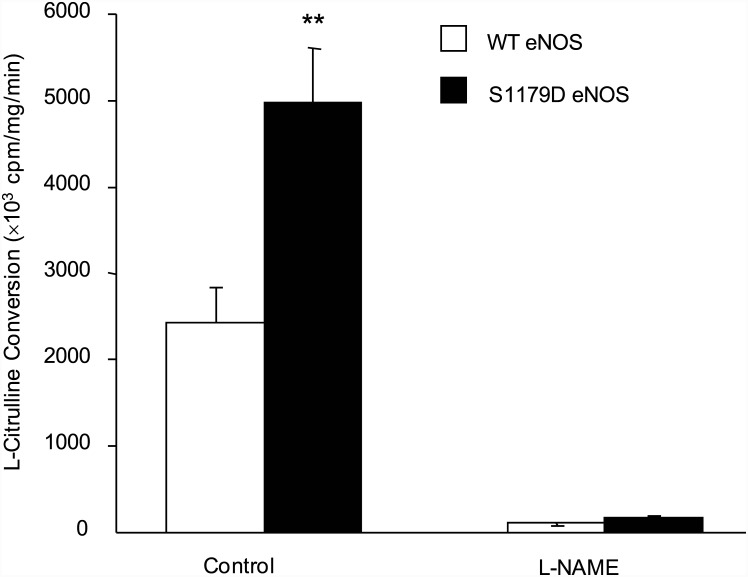
S1179D eNOS has higher rate of NO production than WT eNOS. Enzymatic activity of purified WT eNOS and S1179D eNOS preparations was assayed by monitoring the conversion of L-[^14^C]arginine to L-[^14^C]citrulline. L-[^14^C]citrulline production by S1179D eNOS was significantly higher than WT eNOS in control reactions (**, *p*<0.01,compared with WT eNOS, *n* = 6). No L-[^14^C]citrulline was produced by either WT eNOS or S1179D eNOS in the presence of the direct eNOS inhibitor L-NAME.

Both wild type and S1179D eNOS were expressed and purified from *E*.*coli*. In a culture of 2 liters, approximately 2.5–4.0 mg of eNOS was typically recovered using 2'5'-ADP Sepharose 4B chromatography. Further purification was performed using FPLC. The purity of eNOS was confirmed on SDS gel using Coomassie staining (data not shown). We compared the activities of wild type eNOS (WT eNOS) and S1179D eNOS by measuring the L-[C^14^]arginine to L-[C^14^]citrulline conversion rate. S1179D eNOS exhibited a significant higher L-[C^14^]citrulline production compared with WT eNOS (4968.7±626.6 ×10^3^ c.p.m/mg protein/ min *versus* 2428.6 ± 420.1×10^3^ c.p.m/mg protein/ min, n = 6, *p* < 0.05). These L-[C^14^]citrulline production could be inhibited by eNOS inhibitor, L-NAME (270.9 ± 10.2 ×10^3^ c.p.m/mg protein/ min *versus* 130.6 ± 4.2×10^3^ c.p.m/mg protein/ min, n = 3) (Fig 1). These results confirmed that L-[C^14^]citrulline production was generated from eNOS and eNOS activity increased by phosphorylation.

### S1179D eNOS has more O_2_^-.^–generating activity than WT eNOS

**Fig 2:**
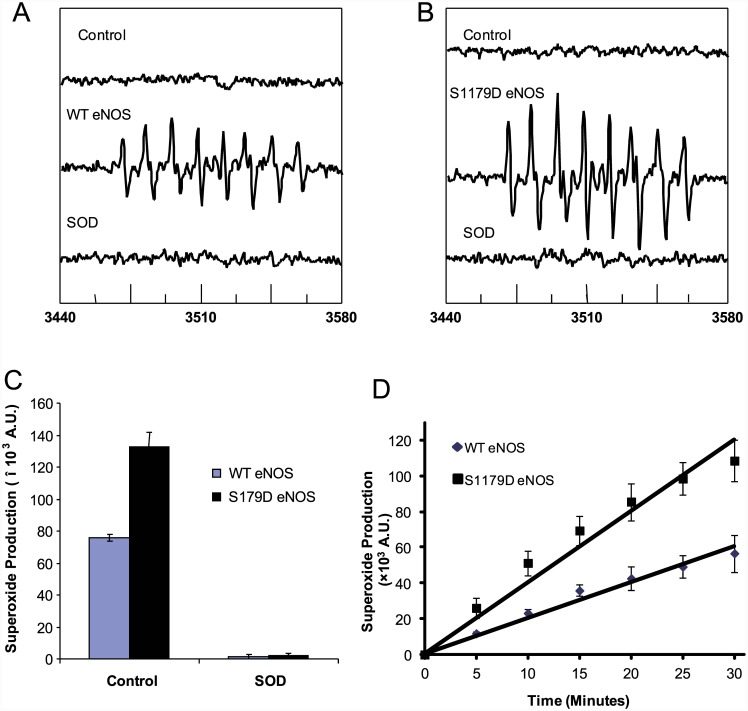
S1179D eNOS has higher rate of O_2_^-.^ production than WT eNOS. EPR spectra of oxygen free radicals generated from eNOS. 300nM eNOS was mixed with the reaction system and followed by EPR described in materials and methods. All results shown are the average of four independent experiments.(A) No signal was observed in the reaction system without enzyme (*top trace*), a prominent spectrum of the DEPMPO-OOH adduct was seen after adding 300 nM WT eNOS (*middle trace*). These signals were totally abolished by SOD (200 units/ml, *bottom trace*). (B) 300 nM S1179D eNOS generated a significantly stronger DEPMPO-OOH adduct EPR signal than WT eNOS (middle trace). These signals were also inhibited by SOD (200 units/ml, *bottom trace*).(C) Total accumulated O_2_^-.^ in 30 minute reactions with WT eNOS or S1179D eNOS, showing that S1179D eNOS has a significantly higher rate of O_2_^-.^ generation than does WT eNOS. Both reactions were blocked by SOD (200 units/ml). (C) Time course of O_2_^-.^ generation by WT eNOS and S1179D eNOS in the reactions described in (C).

Next, we compared the superoxide generation from S1179D eNOS and WT eNOS using EPR spin-trapping experiments. No signals were detected in enzyme-free reaction mixtures containing MEPMPO and NADPH, as well as Ca^2+^/calmodulin (Fig 2A, *Control*). Prominent EPR signals were observed after adding purified WT eNOS (400nM), (Fig 2A, WT *eNOS*). These signals exhibited the characteristic MEPMPO-OOH adduct spectrum(a_N_ = 14.2G,a_H_ = 11.3G,α_H_^γ^ = 1.3G), indicative of trapped O_2_^-^. Compared to WT eNOS, a significantly stronger signal was observed with same reaction system containing 400nM S1179D eNOS,(Fig 2B, S1179D *eNOS*). These signals were totally abolished by addition of SOD (200 units/ml) for both WT eNOS and S1179D eNOS. These results confirmed that the MEPMPO-OOH adduct signals were produced by O_2_^-.^, which was generated by eNOS. S1179D eNOS generated 1.74 ± 0.15 times more O_2_^-.^ than WT eNOS (132321 ± 9196.1 A.U. versus 75953.3 ± 1091.3 A.U. for S1179D eNOS and WT eNOS, respectively; Fig 2C, *p* < 0.01). Kinetic analyses of O_2_^-.^ generation by eNOS showed that S1179D eNOS also had a higher rate of O_2_^-.^ generation than WT eNOS (3617.3 ± 393.0 A.U./min versus 1889.2 ± 347.6A.U./min for S1179D eNOS and WT eNOS, respectively; Fig 2D, *p* < 0.05).

### S1179D eNOS consumes more NADPH than WT eNOS

**Fig 3:**
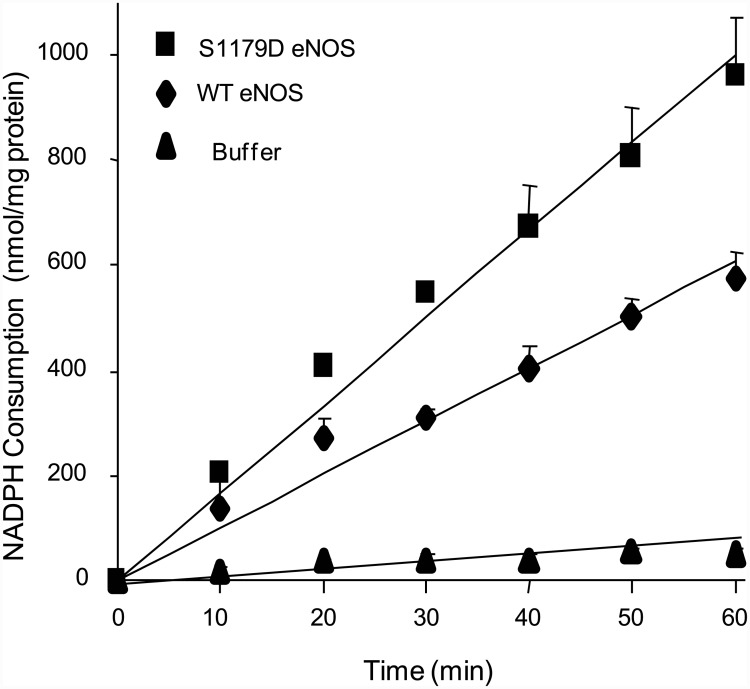
S1179D eNOS has higher NADPH consumption than WT eNOS. NADPH oxidation was monitored spectrophotometrically at 340 nm in the reactions containing 50 mM Tris-HCl, pH 7.4, 50 μM NADPH, 0.5 mM Ca^2+^, 15μg/ml eNOS, and 10μg/ml calmodulin in presence or absence of 300nM eNOS. As shown, there was minimal NADPH consumption in the absence of eNOS, likely representing non-enzymatic oxidation (▲). WT eNOS caused marked NADPH oxidation in the absence of BH_4_ and L-arginine (♦). S1179D eNOS has higher NADPH oxidation rate than WT eNOS (■). Data shown are the mean±S.E. from three independent experiments.

In NOS-catalyzed reactions, the co-substrate NADPH is oxidized and serves as an electron donor for NO or O_2_^-.^ synthesis [4, 21]. Therefore, synchronous NADPH consumption always accompanies O_2_^-.^ generation. To investigate the effect of phosphorylation on the eNOS O_2_^-.^ generation, NADPH consumption by S1179D eNOS and WT eNOS were observed over a 60 minute time course. As shown in Fig 3, there was minimal NADPH consumption in the absence of eNOS, primarily due to non-enzymatic oxidation (52.3 ± 7.8 nmol/mg protein). In contrast, marked NADPH oxidation was seen in reaction mixtures containing either WT eNOS or S1179D eNOS, in the absence of BH4 and L-arginine (577.4 ± 47.7nmol/mg protein and 964.1 ± 106.1nmol/mg protein, respectively; Fig 3). Our measurements of NADPH consumption by eNOS, in the absence of the NO-generating substrate L-arginine, demonstrates that eNOS catalyzes O_2_^-.^ formation activity which is enhanced by serine 1179 phosphorylation.

### O_2_^-.^–generating S1179D and WT eNOS have similar affinities for calcium and calmodulin

**Fig 4:**
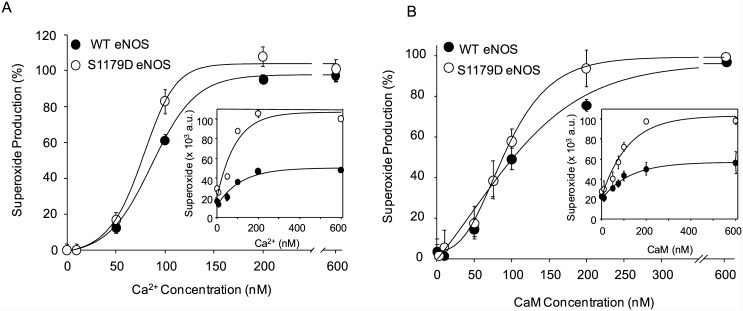
S1179D mutation does not increase affinity of O_2_^-.^-generating eNOS to Calcium and Calmodulin. (A) Calcium-dependent O_2_^-.^ production curves show that S1179D eNOS (*open symbols*) has higher O_2_^-.^ generating activity than WT eNOS (*filled symbols*), but the differences in Ca^2+^ affinity between S1179D eNOS and WT were not significant. (B) Similar to the effect observed with Ca^2+^ affinity, the S1179D mutation also slightly increased O_2_^-.^ generation activity, but did not significantly change eNOS affinity for calmodulin. S1179D eNOS (*open symbols*) and WT eNOS (*filled symbols*). Data shown are mean ± S.E. from *n* = 3–6 independent experiments.

Previous studies showed that the "apparent calcium sensitivity" of eNOS was enhanced in cells expressing either a majority of phospho-eNOS or S1179D eNOS, suggesting that phosphorylation changed the affinity of Ca^2+^/CaM activation [10, 22]. In the present study, we used Electron Paramagnetic Resonance (EPR) to measure calcium and CaM sensitivity changes under conditions where both WT eNOS and S1179D eNOS generate O_2_^-^. As seen in Fig 4A, the calcium sensitivity was slightly increased for S1179D eNOS relative to WT eNOS. However, the EC_50_ values for calcium with WT eNOS and S1179D eNOS were not significantly different (96.7 ± 4.1 and 72.7 ± 4.4nM, respectively, *p* > 0.05, Fig 4A).

To determine if the increased O_2_^-.^ production by S1179D eNOS was attributable to changes in the affinity (or sensitivity) of the enzyme to CaM, we measured O_2_^-.^ production for WT eNOS and S1179D eNOS in different CaM concentrations by EPR. The kinetic data showed that there was a slight left shift in the curve for S1179D eNOS, but little difference in the EC_50_ values for CaM. The EC_50_ values were 103.1 ± 9.5 nM for WT eNOS, and 80.4 ± 7.6nM for S1179D eNOS (*p* > 0.05; Fig 4B).

### BH4 shifts eNOS from O_2_^-.^ generation to NO generation

**Fig 5:**
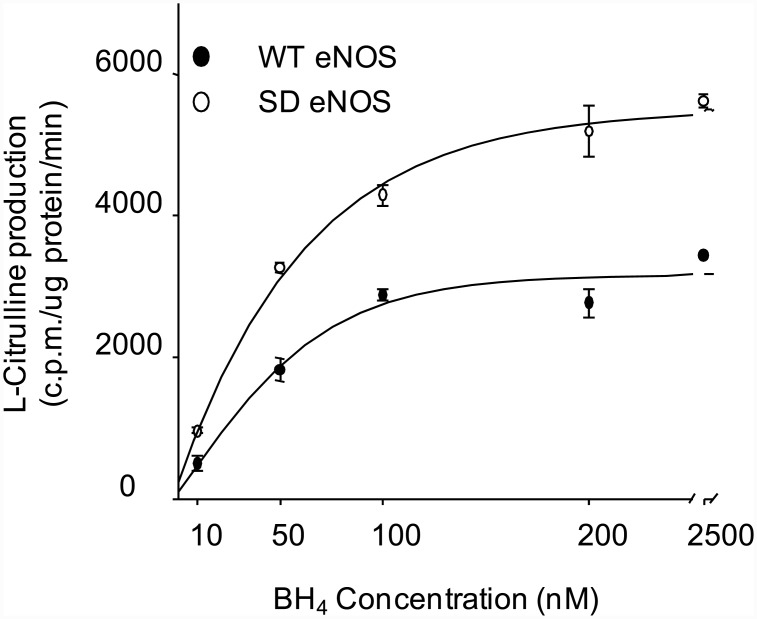
S1179D mutation increased both eNOS activity and affinity to BH4. 1μg purified WT eNOS or S1179D eNOS was added to the reaction system described in Materials and Methods. The indicated amount of BH4 was incubated with the reaction mixture on ice for 10minutes, followed by addition of 2μM L-[C^14^]arginine. The eNOS activity was determined by the conversion rate of L-[C^14^]arginine to L-[C^14^]citrulline. S1179D eNOS had significantly higher activity, and higher affinity to BH4, than WT eNOS. Data shown are the mean ± S.E. from four independent experiments (*p* < 0.05).

Previous reports showed that BH4 plays a central role in regulating eNOS activity [23–24]. Consistent with these reports, both WT eNOS and S1179D eNOS exhibited very low L-arginine/L-citrulline conversion activity under conditions of BH_4_ depletion. BH4 enhanced activities for both WT eNOS and S1179D eNOS dose-dependently. Moreover, our data showed that S1179D had significantly higher sensitivity to BH4 than WT eNOS. The EC_50_ of BH4 to WT eNOS and S1179D were 42.1 ± 2.7 nM and 37.1 ± 1.8 nM, respectively (*p* < 0.05, Fig 5).

**Fig 6:**
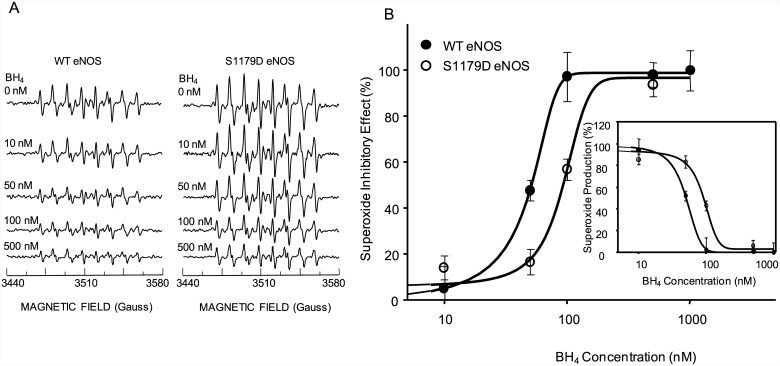
S1179D eNOS is less sensitive to BH4-mediated inhibition of O_2_^-.^ generation than WT eNOS. (A) EPR was performed to determine the effect of BH4 on O_2_^-.^ generation by WT eNOS and S1179D eNOS. For both WT eNOS and S1179D eNOS, a prominent DEPMPO-OOH adduct signal was observed in the absence of BH4 (A, Top panel); As BH_4_ concentration increased, the strength of DEPMPO-OOH adduct signal decreased for both WT eNOS and S1179D eNOS. The experimental conditions were as described in Materials and Methods, with the addition of BH4. (B) Dose-dependence of the BH4 inhibition on O_2_^-.^ generation, showing that S1179D eNOS was less sensitive to BH4 inhibition than WT eNOS.

BH4 plays a vital role in controlling the balance of O_2_^-.^/NO generation by eNOS [4, 25–26]. We tested the affect of serine 1179 phosphorylation on the sensitivity of the superoxide-generating activity of eNOS to regulation by BH4. EPR results showed strong DEPMPO-OOH adduct signal generation by both WT eNOS and S1179D eNOS in the absence of BH4 (Fig 6A top panel). BH4 dose-dependently inhibited O_2_^-.^ generation by both WT eNOS and S1179D eNOS (Fig 6A). Moreover, comparison analyses of the BH4 inhibitory effect on superoxide generation by WT eNOS and S1179D eNOS showed that the S1179D mutant had a significantly lower sensitivity to BH4 than WT eNOS. The EC_50_ values were 55.6 ± 17.5nM for WT eNOS and 103.75 ± 10.9nM for S1179D eNOS, respectively (*p* < 0.05, Fig 6).

### BH4 blocks NADPH consumption by superoxide-generating eNOS

**Fig 7:**
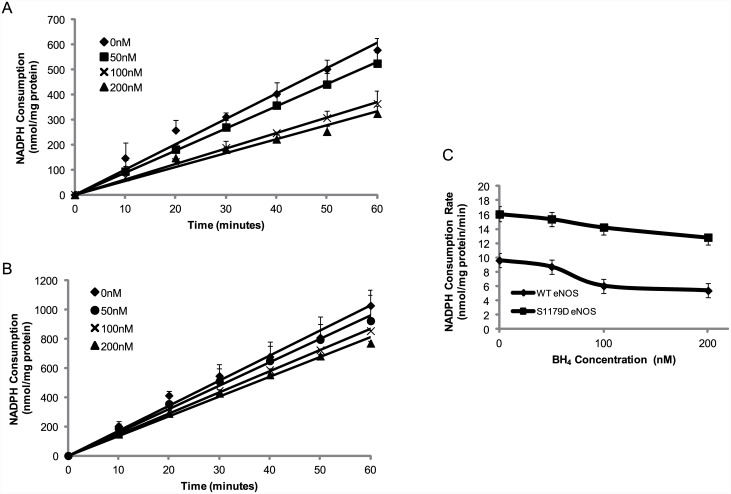
NADPH consumption by eNOS can be blocked by BH4 in absence of L-arginine. 3μg WT eNOS or S1179D eNOS mixed with 600μL reaction system containing 50 mM Tris-HCl, pH 7.4, 0.5 mM NADPH, 0.5mM Ca^2+^, 10μg/ml calmodulin, and BH4 at the indicated concentrations. NADPH consumption was monitored spectrophotometrically at 340nm. (A and B) BH4 showed a dose-dependent inhibitory effect on both WT eNOS and S1179D eNOS, although they had different NADPH consumption rates. (C) S1179D eNOS had a significantly higher NADPH consumption rate than WT eNOS, and was less sensitive to BH4 than WT eNOS. Data shown are the mean ± S.E. from five independent experiments.

As BH4 plays central role in shifting eNOS activity from O_2_^-.^ generation to NO generation, BH4 could be expected to block NADPH consumption in the absence of L-arginine. The NADPH consumption rate for WT eNOS in absence of BH4 was 9.62 ± 0.79 nmol/mg protein/min. BH4 dose-dependently inhibited NADPH consumption of WT eNOS, and 100nM BH4 significantly decreased the NADPH consumption to 6.06 ± 0.52 nmol/mg protein/min (Fig 7A, *p* < 0.05). Similar to the effect of Serine 1179 phosphorylation on the capacity of O_2_^-.^ generation, S1179D eNOS had a significantly higher NADPH consumption rate than WT eNOS in the absence of BH4 (16.1 ± 1.1 nmol/mg protein/min and *p* < 0.05 vs WT eNOS, Fig 7B and 7C). Similar to WT eNOS, BH4 has an inhibitory effect on S1179D eNOS NADPH consumption.100nM BH4 significantly decreased S1179D eNOS NADPH consumption to 14.2 ± 0.08 nmol/mg protein/min (Fig 7B and 7C, *p* < 0.05).

### Loss of cell viability following BH4 depletion was greater in eNOS over-expressing HEK 293 cells than in control 293 cells

**Fig 8:**
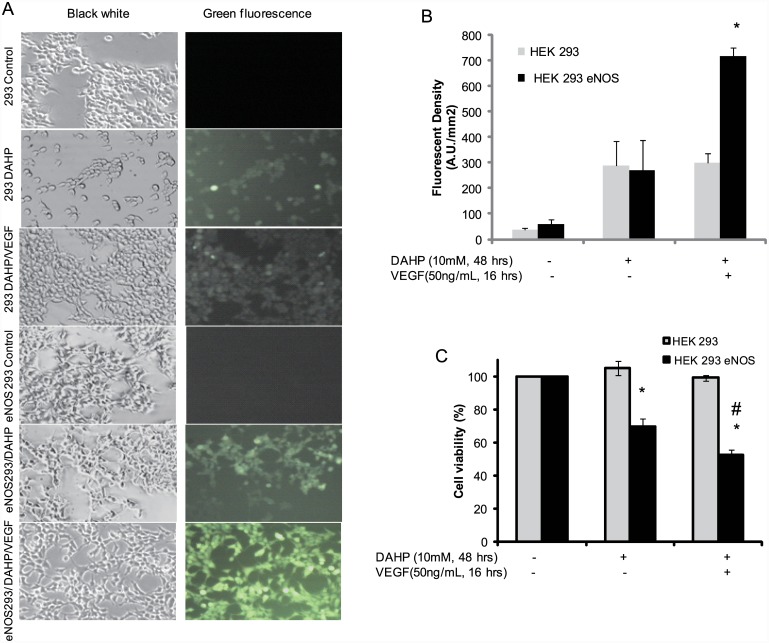
eNOS phosphorylation increased intracellular ROS generation and decreased cell viability in BH4-depleted cells. (A) HEK 293 cells were treated with DMSO or 10mM DAHP for 24 hours to deplete BH4, then transfected with either control vector or WT eNOS plasmid DNA overnight. The transfected cells were incubated with DCF-DA and VEGF as indicated. The fluorescence density was recorded by fluorescent microscopy described in Materials and Methods. (A). Representative fluorescent images are shown. There was no DCF-DA signal in untreated eNOS null HEK 293 cells (first panel, 293 control) or eNOS HEK 293 cells (Fourth panel, eNOS 293 control). A very weak DCF-DA signal was observed in eNOS null HEK 293 in presence of DAHP alone, or with DAHP and VEGF (50ng/mL) together (second panel and third panel, respectively). However, following depletion of BH4 by 10mM DAHP for 48 hours, a prominent DCF-DA signal was observed in eNOS 293 cells (fifth panel). A stronger DCF-DA signal was observed in VEGF-stimulated and BH4-depleted eNOS 293 cells (sixth panel). (B) VEGF-mediated eNOS phosphorylation resulted a significant ROS increase in eNOS overexpressing HEK 293 cells (n = 5 fields, *, *p* < 0.05). (C) eNOS phosphorylation decreased cellular viability in BH4-depleted cells. MTT assays were used to evaluate cellular injury induced by eNOS phosphorylation following BH4 depletion. For eNOS overexpressing HEK 293 cells (Black bars), cellular viability was significantly decreased in DAHP treated cells compared with untreated control cells (HEK 293 eNOS +DAHP vs. HEK293 eNOS control, *, *p* < 0.01). 50ng/mL VEGF caused further decrease of cell viability in eNOS overexpressing HEK293 cells treated with 10mM DAHP (VEGF plus DAHP vs. DAHP alone in HEK 293 eNOS cells, #, *p* < 0.01).

To investigate the effect of eNOS phosphorylation on cell viability under conditions of BH4 depletion [25], intracellular ROS was measured using the cell permeable dye, 2’,7’- dichlorofluorescin diacetate (DCF-DA)[27]. As shown Fig 8, there was no DCF-DA signal in HEK 293 cells which do not express eNOS (eNOS null HEK 293 control, Fig 8A first panel). Following treatment of eNOS null HEK 293 with 10mM DAHP for 48 hours to deplete BH4, low ROS generation was observed in 293 cells (Fig 8A, second panel right). 50ng/ml VEGF for 30 minutes did not significantly increase DCF-DA signal in the control 293 cells (Fig 8A, third panel right).

Similar with eNOS null HEk293 cells, depletion of BH4 by DAHP caused minor increase of DCF-DA signal in eNOS-overexpressing HEK293 cells (Fig 8A fourth and fifth panels). However, in contrast to eNOS null HEK293 cells, phosphorylation of eNOS by 50 ng/ml VEGF for 30 minutes caused a significantly higher DCF-DA signal than control and DAHP treatment alone (Fig 8A sixth panel and Fig 8B, *p* < 0.01).

We next investigated whether or not increased ROS, generated by phosphorylated eNOS, resulted in cell injury following cellular BH_4_ depletion [20]. As shown in Fig 8C, eNOS-overexpressing HEK 293 cells and eNOS null HEK 293 cells were treated with 10mM DAHP for 48 hours to deplete cellular BH_4_. MTT reduction data showed that there was no effect on cellular viability observed for eNOS null HEK 293 cells following cellular BH_4_ depletion. In contrast to the null control cells, BH4 depletion of eNOS-overexpressing HEK 293 cells resulted in significantly decreased cellular viability (*p* < 0.01). Phosphorylation of eNOS in overexpressing cells, using 50ng/ml VEGF, further decreased cell viability (*p* < 0.01). Relative MTT reduction values of the control group, DAHP group, and DAHP + VEFG treated group in eNOS-overexpressing HEK 293 cells were 100%, 69.5% ± 2.2, and 52.2% ± 3.6, respectively.

## Discussion

In this study, we tested the hypothesis that eNOS serine 1179 phosphorylation regulates O_2_^-.^ production, and that serine 1179 phosphorylation can also influence the sensitivity of eNOS to multiple regulatory cofactors. We find that phosphomimetic S1179D eNOS, has significantly higher O_2_^-.^ generating activity than wild type eNOS in absence of BH4 and L-arginine. The S1179D mutation does not significantly alter Ca^2+^ and CaM affinity to eNOS compared to wild-type eNOS. BH4, which inhibits O_2_^-.^ generation by eNOS while simultaneously increasing NO generation, plays a vital role in controlling the balance of NO/ O_2_^-.^ generation by eNOS. In contrast to Ca^2+^ and CaM, we found that serine 1179 phosphorylation significantly decreased the BH4 sensitivity of O_2_^-.^-generating eNOS, while significantly increasing the BH4 sensitivity of NO-generating eNOS.

Consistent with previous observations [10, 28], the S1179D eNOS mutation, which mimics eNOS serine 1179 phosphorylation, increased eNOS superoxide-generating activity 1.74 times over WT-eNOS in our study. The increased activity of phosphorylated eNOS maybe attributable to increasing electron flux at the reductase domain [10]. Serine 1179 phosphorylation induced eNOS activity and enhanced electron delivery efficiency, potentially due to structural changes in eNOS, and altered affinity for its cofactors, including Ca, CaM, BH4 and L-arginine [29].

eNOS is Ca^2+^/CaM-dependent enzyme, thus eNOS activity is regulated by intracellular Ca^2+^ ([Ca^2+^]_i_). For example, shear-stress-mediated eNOS activation indicates that eNOS phosphorylation induced by Akt was a typical Ca^2+^-dependent regulatory mechanism for activation of eNOS [22]. Moreover, Serine 1179 phosphorylation also enhanced eNOS activity in part by increasing its affinity for Ca^2+^/CaM [29–30]. In the current study, Serine 1179 phosphorylation caused an increase of Ca^2+^/CaM affinity to eNOS, relative to WT eNOS, in the absence of L-arginine and BH4, although neither were statistically significant (Fig 4). The discrepancy in the effect of serine 1179 phosphorylation on Ca^2+^/CaM affinity for eNOS, depending on whether we measured NO or O_2_^-.^ production, may be related to the presence or absence of either L-arginine or BH_4_ [4].

In addition to NO, eNOS also generates O_2_^-.^ in absence of L-arginine or BH4 [31]. Recent studies showed that L-arginine, BH4, and HSP90 may play roles in the switch between O_2_^-.^ /NO generation, however, the detailed mechanism has not been defined [4, 32–33]. BH4 plays an independent and central role in balancing O_2_^-.^ /NO generation by eNOS. Depleting or oxidizing BH4 resulted in eNOS dysfunction [4, 33]. In this study, we confirmed that BH4 can dose-dependently inhibit O_2_^-.^ generation from both WT eNOS and S1179D eNOS. Interestingly, our results also indicate that eNOS phosphorylation significantly decreased the affinity of the O_2_^-.^-generating form of eNOS for BH4 (Fig 6). On the other hand, BH4 enhances NO generation from both WT eNOS and S1179D eNOS dose-dependently. In contrast to our findings with O_2_^-.^-generating eNOS, serine 1179 phosphorylation significantly increased the affinity of the NO-generating form of eNOS for BH4 (Fig 5), consistent with previous studies [34]. The divergent effects of BH4 on eNOS activity further define the critical role of BH4 to control O_2_^-.^/NO generation by eNOS. Serine 1179 phosphorylation may induce structural changes in eNOS, which in turn could alter the affinity of O_2_^-.^ generating eNOS for BH4 (Fig 6B).

A previous study showed that S1179D eNOS had a 4-fold higher *K*_*m*_ for NADPH utilization than did wild type eNOS when generating O_2_^-.^ [10]. Our results showed that S1179D eNOS has a significantly higher NADPH consumption rate than WT eNOS (Fig 7C). Consistent with the inhibitory effect of BH4 on O_2_^-.^ generation by eNOS, BH4 also inhibited NADPH consumption by both WT eNOS and S1179D eNOS (Fig 7). eNOS consumes NADPH and generates O_2_^-.^ in absence of L-arginine and BH4, using the same electron transport chains used during NO production. It is therefore reasonable to conclude that Serine 1179 phosphorylation enhances NO generation and O_2_^-.^ generation by the same mechanism [10].

To further define the role of BH4, we investigated the consequences of BH4 depletion on eNOS function and cell viability using an *in-vitro* overexpression model. DCF-DA analysis showed that BH4 depletion results in increased ROS generation in eNOS-overexpressing HEK293 cells. In addition, BH4 depletion results in significantly lower cell viability in eNOS-overexpressing cells compared to controls. Moreover, VEGF-mediated eNOS phosphorylation induced significantly higher ROS in eNOS-overexpressing cells, and significant lower cell viability compared to untreated (minus VEGF) control eNOS overexpressing cells. These results demonstrate the importance of BH4 in regulating both basal and serine 1179 phosphorylated eNOS activity.

In summary, eNOS serine 1179 phosphorylation enhances O_2_^-.^ generation by a Ca^2+/^CaM independent mechanism. In contrast, serine 1179 phosphorylation decreases BH4 affinity for eNOS, and results in an increase in ROS and reduced cellular viability. These novel findings advance our understanding eNOS regulation, and offer new insight into the mechanisms underlying eNOS dysfunction and related clinical disorders.

## Abbreviations

BH4: tetrahydrobiopterin
Ca^2+^: Calcium ion
CaM: Calmodulin
DAHP: 2,4-diamino-6-hydropyrimidine
DCF-DA: 2’,7’- dichlorofluorescin diacetate
DEPMPO: 5-diethoxyphosphoryl-5-methyl-1-pyrroline-*N*-oxide
FPLC: Fast Performance Liquid Chromatography
NO: nitric oxide
NOS: NO synthase
eNOS: endothelial NOS
WT eNOS: wild type eNOS
S1179D eNOS: Serine 1179 aspartic acid eNOS mutant
EPR: electron paramagnetic resonance
L-NAME: *N*-nitro-L-arginine methyl ester
MTT: 3-(4,5-dimethylthiazole)-2,5-diphenyl tetrazolium bromide
O_2_^-.^: superoxide
ROS: reactive oxygen species
VEGF: vascular endothelial growth factor
